# A Port of the Arabian Nights

**Published:** 1874-04

**Authors:** 


### A PORT OF THE ARABIAN NIGHTS.

Bagdad was a notable place in Sinbad's time, and its very name is synonymous with Oriental commercial enterprise, such as it was and is. But even Bagdad and Balsora are beginning to wake up a little under the vivifying influences of the outside world, and may some day or other figure in something less romantic than an Oriental tale, viz., a market report. The French *Journal Officiel* contains some figures with regard to the trade of the former during last year that are not without interest. The skins of oxen constitute the principal exports; the number forwarded last year to Marseilles, Constantinople and Aleppo was 300,000. Marseilles merchants first opened up this trade, and the consequence has been an increase of 50 per cent. in prices, which are now 17 to 24 francs. The skins of goats and sheep from Astrakan are also much sought after. Of 1,000 bales of the former 800 were sent to Marseilles. Of the latter, 70,000 were sent to Russia. The exports of gum, oil seeds, corn and gall were not so satisfactory. The culture of silk—once an important industry—is almost extinct in the province, and what is now exported is received from Bagdad.

